# Ion-Selective Sensors for Orthopaedic Applications: A Systematic Review

**DOI:** 10.3390/bios16060302

**Published:** 2026-05-22

**Authors:** Giorgia Polidori, Andrea Visani, Gianluca Giavaresi, Mauro Serpelloni, Gregorio Marchiori

**Affiliations:** 1 Department of Information Engineering, University of Brescia, Via Branze 38, 25123 Brescia, Italy; mauro.serpelloni@unibs.it; 2 IRCCS–Istituto Ortopedico Rizzoli, Surgical Sciences and Technologies Complex Structure, Via di Barbiano 1/10, 40136 Bologna, Italy; andrea.visani@ior.it (A.V.); gianluca.giavaresi@ior.it (G.G.); gregorio.marchiori@ior.it (G.M.)

**Keywords:** ion-selective sensors, orthopaedic monitoring, prosthetic implants, biocompatible, osteointegration, wearable

## Abstract

Sensors are an established driver of diagnostics and prevention in the medical field, including orthopaedics. Today, the subclass of ion-selective sensors (ISSs) is on the leading edge due to its advantages, enabled by technological advancements in manufacturing, such as miniaturization, precision, accuracy, specificity, a wide measuring scale, ease of use, flexible operating conditions, and measuring speed. While ISSs’ impact on environmental and health fields is already the subject of investigation, it still needs to be analysed specifically in orthopaedics, which is the aim of this Review. A PubMed and Scopus search was performed using the keywords “ion”, “sensor”, “electrodes”, “selective”, “musculoskeletal”, “implant”, “joint replacement”, and “orthopaedic”; after systematic screening, 44 studies were included in the synthesis. First, studies were classified based on the target ion. Only a few papers treated applications specifically in orthopaedics, confirming that ISSs are still largely an unexplored frontier here. However, all of the studies targeted ions with a role also in musculoskeletal pathophysiology, thus relative ISSs could have a potential impact on orthopaedic diagnosis and treatment. Then, when described by the papers, ISSs’ technological solutions were systematically evaluated. Finally, the main ISSs development targets for reaching orthopaedic clinical application were highlighted, including biocompatibility (e.g., implantability), long-term stability, calibration, and validation. Overcoming these challenges will enable ISSs to progress from laboratory prototypes to clinically viable tools, supporting the advancement of next-generation sensorised prostheses, fixation devices, and surgical instruments, and paving the way for predictive and personalised orthopaedic medicine.

## 1. Introduction

Since 2022, sensors have been identified as the next driver of diagnostics and surveillance in orthopaedics trauma, as they are in the larger medical field [1]. Indeed, sensors today have a big role in orthopaedics research and clinics, as testified by numerous review papers on this scope [2,3,4,5,6,7,8,9,10], some of which are reassumed below.

Zhang et al. (2025) focused on advanced piezoelectric materials for orthopaedic medicine, whose pioneering applications also involved sensors [2]. There are characteristics that are specific of piezoelectric mechanisms/materials, overall the sensing of forces/pressure, but also general indications for sensors in the orthopaedic field, as follows: (i) the classification as implantable (e.g., on joint replacements to monitor implant loosening, wear and fracture) or wearable (e.g., for sports medicine), with relative requirements (biocompatibility); (ii) the engineering into nano- and micro-structures; (iii) the need for durability, mechanical performance, high sensitivity, and low power consumption. Future directions in developing novel sensors can be of general interest as well, including the following: (i) monitoring biomechanical changes in bone health and repair processes, and providing real-time support; (ii) monitoring the biochemical environment of post-orthopaedic surgery, such as detecting inflammatory markers or drug release, and ensuring the safety and effectiveness of the recovery process.

Anwar et al. (2025) provided a comprehensive overview of the state of sensor technologies used in bones [3]. The starting point is choosing the target, in this case a bone biomarker. These biomarkers reflect various stages of bone remodelling and are measurable in the blood or urine, offering insights into bone health. Prompt detection of such biomarkers is crucial for diagnosing osteoporosis, bone cancer, and infections, revealing the underlying processes. These biomarkers aid in disease staging and the optimization of treatment. Then, in relation to the target and to its context, the question is which kind of sensor should be used or developed. In this regard, particular emphasis was placed on electrochemical biosensors due to their sensitivity and reliability. Moreover, there is an increasing demand for portable electronic devices capable of translating biological information into readable outputs. However, current sensors face significant limitations and challenges that hinder their widespread application. As a result, further research into biosensors is urgently needed, particularly to enable real-time monitoring with simpler, faster, more cost-effective, and user-friendly methods. Most existing bone biosensors are limited to detecting a single biomarker associated with bone health, but this approach is often insufficient for the accurate and timely diagnosis of bone diseases. Thus, developing multiplex detection methods for multiple analytes is essential to overcome these obstacles, and this work will show that specific ions can be included among those analytes. Multiplex assays offer high sensitivity and require smaller sample volumes. Furthermore, there is a growing need for label-free detection techniques in the near future.

Wang et al. (2023) reviewed the implantable sensors in the musculoskeletal system, useful to collect internal data and to track the condition of the implant [5]. Key application areas include joints, the spine, and fracture management. Future generations of sensors are expected to integrate flexible and biodegradable materials. In addition, there is growing interest in biomaterials with antibacterial surface properties to reduce the risk of infection. Power supply technologies will also continue to evolve to support increasingly sophisticated implants, including systems capable of harvesting energy from human movement and converting it into electrical power for sensors. Currently, multifunctional implantable sensors are a major focus of the research. Beyond providing conventional monitoring feedback, these sensors may also enable treatment optimization based on real-time data. The authors conclude that implantable sensors in the musculoskeletal system are likely to see widespread adoption in the future.

Finally, Viswanathan et al. (2023), discussing sensor-based technology applications in trauma and orthopaedic surgery, highlighted another area of interest that is intra-operative, with sensor-guided instrumentation, which can enhance surgical precision [9].

The aforementioned studies have not mentioned ion-selective sensors (ISSs), where the target analyte, the biomarker, is an atom or molecule with a net electric charge. However, the role of ions was suggested, for example, in [2], where ion channels were said to be protagonists in cellular signalling. Therefore, the hypothesis is that ISSs can play a role in orthopaedics, but the how is not yet defined, which is the aim of this study. Focusing on this sub-class of sensors for potential orthopaedic applications for the first time, starting from defining them and their advantages in relation to the requirements presented above, proceeding from a generic search to revealing the eventual specific clinical needs and potential ISS solutions. Among the various technologies available for chemical and biochemical monitoring, ISSs are a key element due to their ability to provide selective, real-time, and often minimally invasive detection of ionic species. In this review, we adopt the term ISS as a generic term that encompasses both ion-selective electrochemical electrodes (ISEs), and alternative ion-selective detection approaches, such as colorimetric [11], optical [12], and fluorescence-based systems [13]. Although most published data have focused on ISEs, more and more non-electrochemical ISS approaches have emerged that can offer complementary or superior performance in specific applications.

Historically, ISEs have been the most established type of ISS. They are a subclass of electrochemical sensors capable of selectively detecting specific ions present in a sample by establishing of a Nernstian equilibrium at the surface of the sensing membrane [14]. ISEs consist of a selective membrane whose content and composition, especially the ionophore and supporting material, determine the ionic specificity of the sensor. Introduced in the 1930s, ISEs have gradually evolved from bulky laboratory devices to miniaturized solid-state sensors suitable for portable, wearable, and implantable platforms, which show promise for future medical applications [15]. Their high analytical performance, robustness, and relatively low cost have made them the gold standard for ion detection in numerous chemical and biomedical applications. Over time, different types of membranes—glass, liquid, and solid—have been developed, further expanding the possible applications [16]. In addition to electrochemical detection, non-electrochemical ISS systems, including colorimetric and fluorescence-based probes, are also increasingly being studied. These approaches can offer higher spatial resolution, multi-ionic detection, or alternative readings compatible with optical imaging, thereby expanding the range of clinical and research applications [17,18].

Despite the fact that ISSs are already widely used for the analysis of biological fluids, such as sweat, blood, and saliva, their use in more invasive contexts, particularly in post-operative intracorporeal monitoring in orthopaedics, remains a relatively unexplored but highly promising area of research. In this context, ISSs have the potential to provide unprecedented information about local ionic environments: the real-time monitoring of parameters such as pH, density of K^+^, Ca^2+^, Mg^2+^, and other ions at the surgical site could help clinicians in detecting infections, inflammatory processes, or changes in bone integration, thereby improving patient outcomes and enabling more personalized recovery strategies. While these prospects are promising, the integration of ISSs into invasive or implantable settings is still in its early stages, and significant technical and biological challenges must be addressed before widespread adoption can be achieved.

Indeed, in orthopaedic clinical practice, inertial, optical, and pressure sensors for movement and stress analysis are far more diffused than ISSs. Moreover, studies on ISSs explicitly designed for orthopaedic applications are still rare in the scientific literature. Nevertheless, ISSs represent a promising perspective for functional metabolic monitoring, revealing electrolyte imbalance and inflammatory states, and preventing complications, among other things, because of the crucial role of ions. This review, therefore, aims to clarify the terminology and scope of ISSs; to present current ISS technologies and applications with a focus on orthopaedic scenarios; to discuss their metrological performance and potential clinical benefits; and to highlight the open challenges and future directions for research and clinical translation.

## 2. Methods

### 2.1. Eligibility Criteria

Scientific studies were included when dealing with ion-selective sensors (ISSs) targeting ions with a potential role in musculoskeletal pathophysiology and regeneration, both in clinical and pre-clinical (in vitro, ex vivo, or in vivo) research. On the other hand, studies were excluded when dealing with the following: (i) purely dental applications; (ii) ions with no role in orthopaedics as they emerge from the scientific literature; (iii) sensors that are not actually ion-selective.

### 2.2. Search Strategies

**Figure 1:**
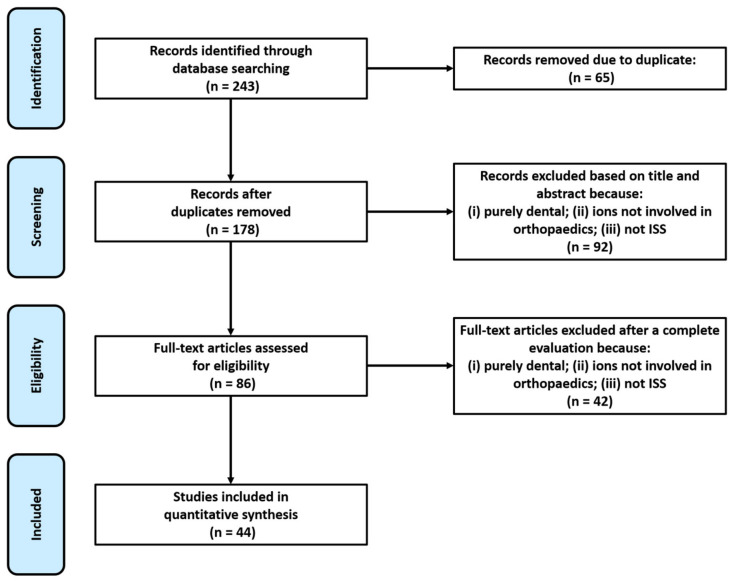
PRISMA flowchart for the eligibility of the studies.

A search was performed on online electronic databases—PubMed and Scopus—on 23 May 2025 with the queries listed in Table 1, which is summarized in the PRISMA flow diagram in Figure 1. The query is the result of a preliminary sensitivity and selectivity analysis that avoids some terms and/or uses others, such as “potentiometric”, “graphene”, “nanostructures”, and “biomedical”. It aimed both at not excluding high-value studies, e.g., on prosthetics and osseointegration, and at identifying a reasonable number of candidates.

Scientific studies from January 2016 to December 2025 (a ten-year range) were included in this review if they met the eligibility criteria (see Section 2.1). Abstracts, reviews, letters, comments to the editor, protocols and recommendations, editorials, and guidelines were excluded, together with scientific studies not written in English.

### 2.3. Study Selection

The achieved lists of studies were first submitted to the Mendeley public reference manager to eliminate duplicates. Studies were then screened by two reviewers (G.M. and G.P.), considering the title, abstract, and eventually full text. Any disagreement in the screening process was discussed by all the authors, and the thought of the majority of the authors was considered the decision-making choice.

### 2.4. Study Assessment

First, all included studies were classified according to their target ion, and potential orthopaedic applications were highlighted, based on the existing literature linking specific ions to musculoskeletal processes and treatments (a single study, or a given ion, could cover multiple areas of application). Then, since ion-selective sensors (ISSs) include both electrochemical ion-selective electrodes (ISEs) and non-electrochemical approaches (optical, colorimetric, fluorescent), we included all types of sensors in the ISS framework. Data on the performance, reliability, and usability of ISSs were extracted whenever available to allow for systematic evaluation. Finally, the results were synthesized in terms of orthopaedic scope, level of ISS validation, and pre-clinical or clinical phase.

## 3. Results

### 3.1. Selection of the Studies

The initial literature search recovered 243 studies. Of those, 175 studies were identified using Scopus, and 68 were found on PubMed. Aiming to eliminate duplicates, articles were run through Mendeley v1.19.8, removing 65 studies. The remaining articles—i.e., 178—were then screened considering their title and abstract, resulting in 92 studies discarded. The full text of the leftover 86 articles was then reviewed, allowing for the removal of 42 additional studies due to non-compliance with the eligibility criteria. The remaining studies—i.e., 44—were, finally, investigated. The overall process is detailed in Figure 1, which maps out the number of records identified, included, and excluded.

### 3.2. Classification of the Studies

The target ions, potential orthopaedic applications, and targeting ISS studies are listed in Table 2. In the following sections, for each ion, the connection ion–orthopaedics and the main relevant ISS studies will be presented. One of the most widespread ISS applications relies on ion quantification within biofluids (sweat, plasma) to monitor human health in terms of hydration state, muscular exercise, and different types of diseases (ex vivo/in vitro). Research is rapidly expanding to other areas, including the monitoring of bone status and skeletal studies (in vivo) [19]. Therefore, for the same ion, first the ex vivo/in vitro investigations will be presented, then the eventual in vivo ones.

### 3.3. Calcium, Sodium and Potassium Ions (Ca^2+^, Na^+^, K^+^)

The calcium ion (Ca^2+^) has a fundamental role in the musculoskeletal system: Ca^2+^ is important for bone mineralization, it is a messenger to control skeletal muscle contraction [65], and is crucial for synaptic transmission and other processes in the nerves [66]. Pasquarelli et al. (2022) reported the innovative application of high-sensitivity microelectrodes for tracking small changes in Ca^2+^ concentration into the lipid bilayer of liposomes, which could not be assessed using common Ca^2+^ selective electrodes, supporting the hypothesis of selective Ca^2+^ transport in the mineralization initiation process that occurs in matrix vesicles (in vitro investigation) [20]. Dedic et al. (2019), with a study on mice, measured ex vivo by ISE the Ca^2+^ fluxes at the bone–extracellular fluid interface and argued that the fastest response in the continuous temporal hierarchy of events addressing a disruption in plasma Ca^2+^ homeostasis is independent of the parathyroid hormone and its associated signalling through its receptor in osteocytes [21]. Hendrianingtyas et al. (2022), in a clinical study, identified a high Ca–P ratio as an osteopenia risk factor in women with central obesity, measuring serum Ca^2+^ levels ex vivo by ISE [22]. Yu et al. (2024), in a study on rats, introduced a new implantable biodegradable fibre calcium sensor that can monitor the fluctuations of Ca^2+^ in a 4-day lifespan in vivo and biodegrade in 4 weeks [25].

The study of Jiang et al. (2020) deserves a mention in itself, as it proposed a strategy based on antimicrobial organic agent release for a Ca^2+^-ISE, tested in vitro against bacteria [23]. Indeed, a sensor is an implant in itself when designed for inside-the-body applications, thus peri-sensor infections should be avoided. Also, sodium and potassium ions (Na^+^, K^+^) have a link with bone pathophysiology [31,67,68], are essential for muscle function [68], and are crucial in neuron function [69]. Therefore, the ISEs designed and implemented in [27,28,29,32] should be taken into consideration (see Section 3.9). In particular, Tsou and Cheng (2024) developed miniaturized Na^+^/K^+^ ISEs with superior sensing performance for real-time monitoring in portable clinical applications [27], and Ozer et al. (2022) developed ISEs for the multiplexed detection of Na^+^ and K^+^ in urine and artificial sweat samples (wearable device) at clinically relevant concentration ranges [32]. In another study, Wildner et al. (2020) presented a K^+^ ISE with anti-biofouling properties [29]: the ISE was found not only to operate successfully in buffer solutions, but in artificial cerebrospinal fluid, indicating that it could be adapted for use in living nerve bundles. The sensor’s very good mechanical properties in combination with its anti-biofouling properties render it an excellent potential tool for the chemical monitoring of neural and other physiological activities using implantable devices [29]. Bao et al. (2019) fabricated flexible K^+^/NH_4_^+^/Ca^2+^ selective sensors and tested them with a mixture of interference ions concentration in artificial saliva, confirming their high selectivity in a mixture of interference ions and expanding the possibility of wearable sensors for real-time monitoring in biological or medical applications [24]. Cánovas et al. (2019) assessed the toxicity of widely used ISEs targeting some of the most common body ions (K^+^, H^+^, Ca^2+^, Na^+^, NH^4+^) through in vitro assessments of cell viability, proliferation, and adhesion using human fibroblasts, with the results influenced by the specific ISE configurations [32]. The authors emphasized the importance of conducting biocompatibility evaluations from the earliest stages of sensor development. This approach facilitates compliance with the requirements for future on-body applications and large-scale in vivo validation prior to device commercialization [33]. The study by Yang et al. (2022) is of particular interest, even if indirectly connected to orthopaedics: electrophysiology and neurochemicals, such as Ca^2+^, K^+^, and Na^+^, on the cerebral (motor) cortex can synergistically reflect the neurophysiological states [26]. The authors developed a flexible multifunctional electrode to simultaneously record electrocorticography and extracellular ions of Ca^2+^, K^+^, and Na^+^ on the surface of the cerebral cortex; they tested it in vitro and in vivo by attaching it to the surface of rats’ cerebral cortex [26].

### 3.4. Fluoride Ion (F^−^)

Regarding fluoride (F^−^), it has an influence on the musculoskeletal tissues. Excess fluoride ions are strongly attracted to the cationic calcium present in bones, which results in skeletal fluorosis; as such, fluoride concentration should be within a certain range in drinking water. Workeneh et al. (2019) developed a cost-effective fluoride removal technique to remove the surfeit fluoride from water and tested its absorption capacity in a fluoride solution by a F^−^ ISE [30]. In the same field, Swami et al. (2022) developed an ISS strategy for rapid and effective analytical sensing of F^−^ in an aqueous medium [34]. Kodsup et al. (2022) measured F^−^ concentrations in drinking waters using the ISE method, categorized individuals by F^−^ concentrations, and demonstrated the effect of F^−^ exposure on bone quality [35]. Those ISEs should be tested in more complex solutions for ex vivo or in vitro—i.e., on body fluids or in cell cultures—applications.

Mirjalili et al. (2020) developed nanosized biocomposites with properties close to the bone and good biocompatibility for use in dentistry, maxillofacial bone defects, and orthopaedics as a bone constructor, and used an F^−^ ISE to measure the concentration of released F^−^ in a simulant solution [37]. Oikawa et al. (2018), in a clinical study, measured the serum ionic fluoride (SIF) concentration by ISE (ex vivo) and concluded that SIF may be useful for diagnosing bone union [38]. Ossification of the posterior longitudinal ligament (OPLL) is a progressive disease that causes spinal canal compromise and serious neurological sequelae in advanced cases. Reddy et al. (2018) studied the association of OPLL with fluorosis in a clinical study measuring the 24-h urine F^−^ level by the ISE method (ex vivo); fluorosis was associated with a higher incidence and severity of OPLL [39]. Fluoride toxicity could induce tendinopathy and musculoskeletal toxicity. Bidu et al. (2025) reported a case where the use of ciprofloxacin (a fluoroquinolone antibiotic widely used in clinical practice) induced a high urinary F^−^ concentration—measured ex vivo by ISE—associated with tendinopathy and peripheral neuropathy [40].

Definitely, F^−^ plays a significant biological role in the human body, participating in the binding of magnesium, calcium, phosphorus, and in the mineralization of hard tissues. For this reason, Ciosek et al. (2019) measured the F^−^ concentration by an ISE in a sample of infrapatellar fat pad tissue collected from each patient (ex vivo) in a clinical study on subjects who had undergone total knee replacement [41].

### 3.5. Hydrogen Ion (H^+^)

pH measures the H^+^ concentration in a solution, and it is a crucial factor for the correct functioning of numerous chemical reactions in the human body. ISEs were used by Cordoba et al. (2018) [42] and Gnedenkov et al. (2016, 2019) [43,44] for evaluating the effect of metal implants’ corrosion on the pH of phosphate-buffered saline solution, thus proving ex vivo what could happen in vivo.

Orthopaedic implants are highly susceptible to peri-implant sterile inflammation or microbial infections, Ivanko et al. (2022) developed an ISE on a titanium alloy support for the detection of peri-implant pH changes to enable the early detection of inflammation/infection [45]. The physiologically relevant pH changes were measured by the ISE in the synovial fluid acquired from patients (ex vivo) during orthopaedic surgery (infected or not, total knee or hip endoprosthesis) [45].

pH is a critical indicator of bone physiological function and disease status; however, non-invasive and real-time sensing of bone pH in vivo has been a challenge. Therefore, Li et al. (2020) synthesized a bone pH sensor and tested it on rat bones in vivo, demonstrating that their solution has the potential for minimally invasive and real-time in vivo bone pH sensing in pre-clinical studies of bone healing, metabolism, and cancer mechanisms [46].

### 3.6. Zirconium, Nikel, Chromium, Aluminium, Cobalt, and Magnesium Ions (Zr^4+^, Ni^2+^, Cr^6+^, Al^3+^, Co^2+^, Mg^2+^)

Orthopaedic implants—such as endoprostheses, fixation devices, screws, and scaffolds—can release ions into the body because of a functional coating, degradation, or wearing, with a potential local and/or systemic effect. Metal detection in fluids is the object of various standards for implants characterization (e.g., [ISO 10993] [70], [ISO 17853] [71], [ISO 10271] [72]).

Zirconium-based alloys have received increasing attention as implant candidate materials due to their superior biocompatibility, good mechanical properties, and compatibility with magnetic resonance imaging [73]. However, the zirconium ion (Zr^4+^) is harmful to human health, and this is the reason why Liu et al. (2024) developed a highly sensitive ISS method to conveniently detect Zr^4+^ [47]. Singh et al. (2024) developed a rapid-sensing Zr^4+^ ISS platform, which furthermore exhibited in vitro antibacterial and antioxidant properties [48].

Nickel-Titanium (Ni-Ti) shape memory alloy implants have increasingly become the first choice for fracture and massive bone defects after orthopaedic bone tumour surgery [74]. However, several biological factors can influence nickel corrosion and metal ion release, potentially toxic to physiological systems. Zhang et al. (2021) developed a sensor targeting Ni^2+^ with high sensitivity, excellent specificity, and fast response, which was compatible with cell imaging (in vitro), and thus was expected to be a useful probe for Ni^2+^ in broad biological application [50]. Saikrithika et al. (2022) interrogated and visualized in situ Ni^2+^ release with a new and sensitive approach relevant to biological toxicity [49].

Other metals used in implants are cobalt, chromium, tantalum, titanium, vanadium, aluminium, and magnesium. Metals in contact with biological systems undergo corrosion, which leads to the formation of metal ions, and may activate the immune system (i.e., hypersensitivity) [75]. Chromium, as Cr^6+^, is very harmful due to its high carcinogenic and mutagenic properties. Ebrahim et al. (2020) were successful in recognizing Cr^6+^ in real water samples [51], while Ming et al. (2019) used a different sensor with analogous finalities [52]. Bartwal et al. (2020) designed and synthetized two different probes for revealing aluminium and cobalt ions (Al^3+^, Co^2+^) in semi-aqueous media [53]. Again, those ISSs should be tested in more complex solutions for ex vivo or in vitro applications—i.e., on body fluids or in cell cultures.

Last, the magnesium ion (Mg^2+^) is a regenerative cue especially for the bone tissue environment. Wang et al. (2022) tested bone grafts, eventually enriching them with magnesium, by monitoring their Mg^2+^ releasing profile by ISE in an aqueous solution [54].

### 3.7. Argentum and Cuprum Ions (Ag^+^, Cu^2+^)

Any changes in the trace element content manifesting in a deficiency or excess of metals in the human body may impair the functioning of the skeletal and articular system and predispose the body to the development of diseases, such as osteoporosis. Thus, despite silver and cuprum ions (Ag^+^, Cu^2+^) antibacterial properties being well known and that they can be used to avoid infections on the implant surface [76], if their concentration is too high, they can have harmful effects (e.g., cytotoxicity). Yaghoubi et al. (2024) developed a highly selective, low-detection-limit Ag^+^ sensing platform [55]. Mousavi et al. (2024) designed a cost-effective, short response time, selective, sensitive, and usable at different pH, sensor for detecting Cu^2+^ in biological samples [60]. Jain et al. (2023) reported a novel, selective, and sensitive detector of Cu^2+^ in nanomolar concentration [56]. Similarly, the Cu^2+^ sensors proposed by Li et al. (2016) [57], Saadeh et al. (2022) [58], and Habib et al. (2019) [59] are remarkable.

### 3.8. Other Ions and Electrically Charged Molecules

In the study of Kaewnok et al. (2025), it was reported that gold complexes and nanoparticles have been utilized in the treatment of arthritis, but at the same time, gold ions (Au^3+^) can manifest adverse impacts on human health [61]. For these reasons, the authors synthesized and applied a sensor for Au^3+^ monitoring in near-perfect aqueous media; the sensor demonstrated high potential for tracking Au^3+^ levels also in human cells [61].

Several orthopaedic pathologies involve inflammatory processes, such as arthritis. In them, various ions have a role, H^+^ included (pH), as cited in Section 3.5. Indeed, Dong et al. (2018) developed a sensor with an ion-based proximity quenching mechanism for revealing human/mouse proteins and antibodies, also present in joint inflammations (e.g., IgG), and tested the sensor in vitro [62].

Coming to implants, phosphate coatings can be used on metal scaffolds to increase their biomimicry towards bone, and thus promote osteointegration: Trybuś et al. (2017) deposited phosphate coatings on porous steel scaffolds and analysed the content of Ca^2+^ and PO_4_^3−^ (phosphate ion) after the coating dissolution in a nitric acid solution [63].

Finally, mention is given to the study of Pandey et al. (2017): they developed a urea biosensor with ammonium (NH^4+^)-ISE [64]; in hip fracture patients, admission serum urea is an independent and valuable predictor of hospital outcomes, in particular, mortality [77]. Therefore, the monitoring of fracture patients by urea analysis could benefit from the advantages of an ISS.

### 3.9. Technologies and Applications of ISSs

**Figure 2:**
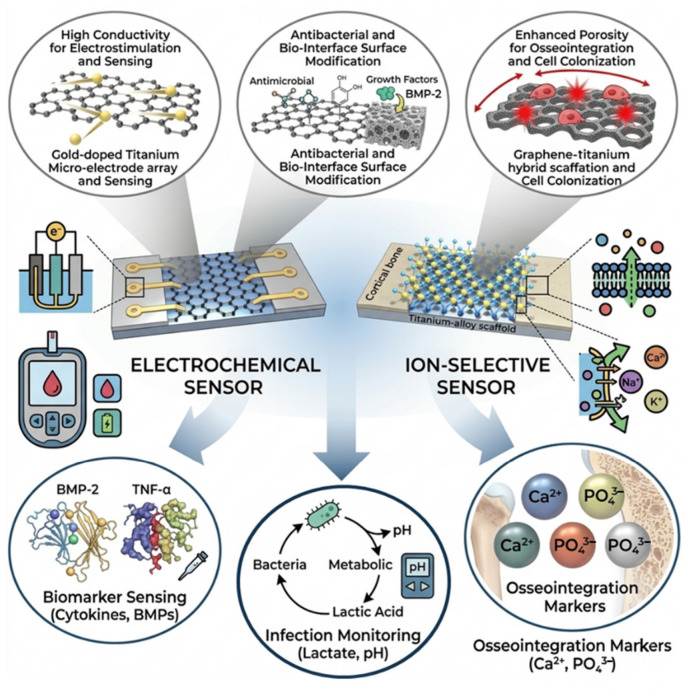
Schematization of Ion-Selective Sensors with electrochemical electrodes—highlighting the mechanism of ion sensing—and of potential applications. Electrochemical sensors are used to detect molecules larger than simple ions, such as BMP-2, TNF-alpha, or cytokines—molecules that play a crucial role in the musculoskeletal physiopathology and show a strong clinical interest. One example is the pH sensor, which allows for the measurement of the hydronium ion. This is relevant in the context of prosthetics, where the sensor could be integrated for monitoring infections (often caused by the formation of a bacterial biofilm on the prosthesis surface) before symptoms become clinically evident. Bacteria that colonize the implant consume nutrients and produce waste products. Many common pathogenic bacteria found in orthopaedic infections (such as *Staphylococcus aureus*) produce lactic acid as a by-product of their anaerobic metabolism. The accumulation of lactic acid causes a drastic decrease in the local pH (acidification of the environment around the implant). This is where the device technology comes into play, through the detection of H^+^. A pH ion-selective sensor is designed with a membrane sensitive exclusively to hydrogen ions (H^+^). Since pH is a measure of the concentration of these ions, the sensor reveals the acidification caused by the bacteria. In addition to pH, ion-selective sensors monitor ions such as calcium (Ca^2+^) and phosphate (PO_4_^3−^). In case of infection, the normal process of osteointegration (formation of new bone) is disrupted. If the sensor detects that the levels of these ions do not follow the expected trend for healing, it can translate the chemical concentration into a digital electrical signal that can be monitored by the physician. These sensors are not bulky separate components, but can be integrated into the implant itself, thanks to advanced materials such as graphene and titanium. Graphene (shown as a hexagonal mesh) acts as a highly conductive interface, allowing the sensor to be extremely small (miniaturized) and sensitive. Additionally, the surface can be engineered to be both antibacterial and sensitive, creating an environment that combats infection while simultaneously monitoring it.

As illustrated in Section 3.3, Section 3.4, Section 3.5, Section 3.6, Section 3.7 and Section 3.8, ion-selective sensors (ISSs) and ion-selective electrodes (ISEs) in particular (Figure 2) have been studied in a wide range of contexts with orthopaedic potential, from bones and muscles to implants, nerves, inflammatory processes, and body measurements. The effective translation of these technologies into clinical practice depends on advances in electrode design, materials, integration strategies, and complementary analytical approaches. Based on the following classification, we grouped the selected studies into three main categories to reflect their technological focus and degree of metrological characterization.

**Figure 3:**
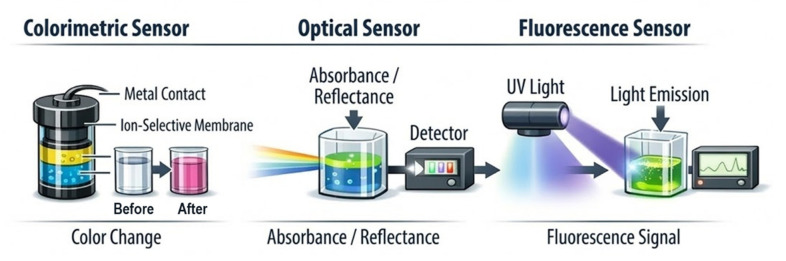
Schematization of non-electrochemical sensors, highlighting the various detection systems. For colorimetric sensors, they exploit a colour change due to the formation of a complex between an ion and an indicator. Examples where colorimetric ISSs are used include Ca^2+^ (crucial for bone mineralization), Mg^2+^, F^−^ (fluorosis, bone quality, release from materials), metals released from implants (Ni^2+^, Co^2+^, Cr^6+^, Al^3+^, Zr^4+^), and physiological ions, Na^+^, K^+^, PO_4_^3−^ (phosphate, mineralization). For optical sensors, they measure how light is absorbed or reflected by a membrane or by a material sensitive to the ion. The ions detectable with optical sensors are usually ions that form coloured complexes, modify the refractive index, or alter the optical state of a membrane. Potentially for orthopaedics, Ca^2+^, Mg^2+^ (mineralization), Na^+^, K^+^ (cellular homeostasis, muscle, nerves), F^−^ (fluorosis, bone quality), and metals from implants, such as Ni^2+^, Co^2+^, Cr^6+^, Al^3+^, Zr^4+^, PO_4_^3−^, can alter the optical absorption of immobilized indicators or of sensitive materials. For fluorescence sensors, the most common target ions are Ca^2+^ (e.g., Fura-2, Fluo-4), Mg^2+^, heavy metals (Pb^2+^, Hg^2+^, Cd^2+^), transition metals (Fe^3+^, Cu^2+^, Co^2+^), anions like F^−^, CN^−^, ClO^−^. Ca^2+^ central for mineralization, cell signalling, and in vivo monitoring (e.g., Yu et al., 2024, which discusses a biodegradable sensor [25]). In fact, it is one of the ions best suited to fluorescence.

The first group comprises non-ISE ISSs, which extend the concept of ion detection beyond classical electrochemistry (Figure 3). These include optical, colorimetric, and nanomaterial-based systems, as well as emerging field-effect transistor (ISFET) platforms. Such technologies exploit fluorescence quenching, absorption shifts, or ion-sensitive surface modifications to achieve ion recognition.

Compared to traditional electrodes, these systems offer clear advantages in terms of miniaturization, multiplexing, and even visual or label-free detection, making them attractive for point-of-care and implant-integrated applications. Unlike conventional ISEs, most of these platforms are not evaluated using standard electrochemical parameters (e.g., slope, stability), but many still report key metrological indicators, such as detection limits, linearity, and pH operating range.

The metrological parameters available for the non-electrochemical sensors included in this review are listed in Supplementary Table S1. Since these platforms exhibit significant heterogeneity in the data reported in the literature, the table lists only the indicators that are actually available (LOD, linear range, response time, operating pH, and selectivity), indicating “NA” when the information is not provided by the original authors. For example, fluorescence and colorimetric sensors often exhibit limits of detection (LODs) in the μM–nM range [34,47,48,49,55,56,60], with linear ranges corresponding to biologically relevant ionic concentrations in tissue and fluid models [51,52,61]. Several systems demonstrate stable responses in physiological pH ranges, typically pH 5 to 8 [46,61,62]. These values, although not directly comparable to Nernst slopes, provide meaningful indicators of sensitivity and analytical selectivity under biological conditions. Numerous studies have focused on flexible transistor-based sensors, which combine ionic selectivity with advanced electronic architectures. Bao et al. (2019) introduced a flexible 3D-printed ISFET platform to detect NH^4+^, K^+^, and Ca^2+^ in artificial saliva, reporting slopes close to the theoretical Nernst values (NH^4+^ ≈ 98 mV/decade, K^+^ ≈ 104 mV/decade, Ca^2+^ ≈ 42 mV/decade) and improved stability with AgCl-modified references [24], confirming that classical ISE principles remain relevant for FET-based devices. Similarly, Yang et al. (2022) explored how nanowire geometry and back-gate configuration affect the sensitivity of ion transistors, observing “super-Nernstian” behaviour but without evaluating reproducibility or long-term stability [26]. Other studies have emphasized environmental robustness and standardization. For example, Li et al. (2016) developed reproducible artificial saliva matrices for inter-laboratory repeatability [57], while Zhang et al. (2021) analysed wearable sensory patches in which movement, sweat composition, and variable humidity introduce non-negligible metrological uncertainty [50].

Finally, optical and colorimetric platforms offer additional versatility. Bartwal et al. (2020) developed fluorescent dyes for Al^3+^ and Co^2+^ with LODs down to 10^−8^ M, demonstrating high selectivity (no interference from other cations) and complete reversibility after EDTA addition, thus validating their reusability as a “secondary” probe [53].

The second group includes studies in which ISEs are not the main focus of innovation but are used as analytical or diagnostic tools. In these cases, commercially available electrodes or established configurations are used to monitor ionic species in biological fluids, tissues, or implant materials. Since the electrode is considered to be a validated analytical tool, a complete metrological characterization is rarely provided; instead, the emphasis is placed on biological results or material characterization. Within this group, some studies have applied ISEs to biomaterials and implant studies for monitoring the release of silicon and fluoride from fluorapatite–forsterite nanocomposites [37], for the use of the ion-selective electrode technique (SIET) to map local distributions of Mg^2+^ in allograft bone and link them to osseointegration [54], or for the evaluation of localized corrosion in Mg implants and of protective coatings [42,43,44]. Similarly, phosphate coatings on titanium scaffolds have been evaluated by measuring the Ca^2+^ and PO_4_^3−^ deposition, but without detailed metrological data [63]. Other studies have used ISEs in clinical or diagnostic settings. These include the assessment of urinary fluoride as a diagnostic marker for fluorosis [39], the monitoring of serum fluoride after spinal fusion [38], and calcium fluxes at the bone–plasma interface studied with SIET in ex vivo models [21]. A recent case report by Bidu et al. (2024) described ciprofloxacin-induced musculoskeletal and neurological toxicity associated with elevated urinary fluoride levels measured by an ion-selective electrode, highlighting the diagnostic potential of ISEs in detecting systemic fluoride imbalance related to drug metabolism [40]. Hendrianingtyas et al. (2022) applied commercial Ca^2+^ electrodes in a clinical study on osteoporosis, emphasizing diagnostic accuracy (ROC analysis) rather than electrode performance [22]. Similarly, an analysis of the infrapatellar fat pad after total knee arthroplasty combined ion-selective electrodes for F^−^ with the ICP methods for Ca, Mg, and Pb [41]. Finally, some studies have illustrated non-clinical analytical uses of ISEs, such as monitoring fluoride in environmental matrices with hydroxyapatite adsorbents derived from eggshells [30] or electrochemical studies of crystallization processes (CaOx) using functionalized carbon nanostructures [36]. Overall, these contributions have illustrated how ion-selective detection has already been integrated into biological, material, and clinical workflows. However, the reporting of metrological characteristics remains fragmentary, with only occasional references to operating ranges (e.g., pH 7.5 in [20]), slopes and linearity (−57/−58 mV over 0.3–15.5 mg/L in [35]), or diagnostic thresholds (urinary fluoride cut-off at 1.6 mg/L in [39]).

The third group (Supplementary Table S2) includes studies dedicated to the development and metrological characterization of ion-selective electrodes (ISEs) and ion-selective membranes (ISMs). These works represent the technological core of ion detection, focusing on the fabrication, optimization, and systematic evaluation of innovative detection platforms. In this category, the classic performance parameters—slope, detection limit, linear range, response time, stability, pH operating range, and reproducibility—are explicitly reported and experimentally optimized. Several distinct technological strategies have emerged: early studies explored nanostructured solid-state ISEs to improve electrical conductivity and long-term stability [25,27], followed by the development of potassium-selective membranes incorporating crown ether derivatives (BME-44) within plasticizer-free silicone matrices, where covalent bonding improved selectivity for K^+^ over Na^+^ and significantly reduced ionophore leaching [28]. Another study examined Ca^2+^, K^+^, and Na^+^ ISEs, explicitly addressing cross-interference from Mg^2+^ that involved flexible multifunctional electrodes based on carbon nanotube arrays to record electrophysiology and ions in the cerebral cortex in real time (in vivo), where the interaction of Ca^2+^ is crucial [26].

Other contributions have introduced polymer-anchored ionophores and carbon paste electrodes functionalized with Schiff base ligands, resulting in selective sensors for Cu^2+^ with detection limits down to 10^−8^ M and fast response times of a few seconds [58]. Parallel developments have focused on antifouling and biocompatible interfaces, such as iridium oxide microsensors coated with antibiofouling layers for the detection of K^+^ in physiological media [29] and Ca^2+^-selective membranes doped with 6-chloroindole that exhibit long-lasting antibacterial activity without compromising metrological performance [23]. From a translational perspective, several studies have incorporated the biocompatibility and cytotoxicity testing of ion-selective membranes to enable wearable and implantable applications [33]. Hybrid biosensor configurations have also emerged, such as urea biosensors, that couple silver-polypyridine nanocomposites with immobilized urease to generate ammonium ISE transducers [64]. Finally, recent advances have included the development of smart potentiometric films capable of simultaneously detecting pH and reactive oxygen species (ROS) through non-biofouling poly(2-methyl-2-oxazoline) coatings, a promising approach for the early diagnosis of peri-implant inflammatory processes [45].

Although most of these contributions remain in the experimental or pre-clinical phase, they collectively establish quantitative benchmarks and define the current technological limits of ionic detection in orthopaedics. The systematic distinction between metrologically characterized ISEs, analytically applied electrodes, and emerging non-electrochemical ISSs clarifies where reliable quantitative data are available, where ion-selective detection is already integrated into biological or clinical workflows, and where innovative transduction strategies are expanding the field beyond classical electrochemistry. Taken together, these studies underscore the fundamental importance of antifouling and biocompatible design, long-term stability, and integration with implant materials as prerequisites for clinical translation. At the same time, they demonstrate how complementary detection approaches—optical, electronic, or hybrid—can enhance versatility and enable multifunctional, minimally invasive monitoring systems. Ultimately, this framework provides a comprehensive and critical overview of ion detection in orthopaedics, highlighting the strategies that balance metrological performance, biocompatibility, and clinical usability, and outlining a coherent path toward future implantable and regenerative diagnostic technologies.

### 3.10. Synthesized Findings

Following the data collected from the previous sections and Table 2, ISS (i.e., studies) grouping can follow a clinician’s classification as follows:

- “ISSs for assessing Bone (Muscle/Nerve) health and metabolism” reached validation at the implantable level in the case of Ca^2+^, K^+^, and Na^+^ targets, while at the in vitro/ex vivo level in the case of F^−^, pH, and NH^4+^ targets;
- “ISSs for monitoring implant wear and corrosion” reached validation at the in vitro/ex vivo level for Zr^4+^, Ni^2+^, and Cu^2+^ targets, while at the bench level (i.e., non-biological samples) for Cr^6+^, Al^3+^, Co^2+^, Mg^2+^, Ag^+^, and Au^3+^ target;
- “ISSs for diagnosing peri-implant infection” reached validation at the implantable level in the case of pH, while at the in vitro/ex vivo level for immunoglobulins;
- “ISSs for on-body measurements” reached validation at the in vivo level for K^+^ and Na^+^ target, while at the in vitro/ex vivo level for Ca^2+^, pH, and NH^4+^ target.

Only ISE technology appears implantable, as non-ISE sensors could not be read from outside the body.

Of the included studies, four (9%) are pre-clinical (on animals), among which one is an ex vivo study [21] and three are in vivo “implantable” studies [25,26,46]; seven studies (16%) are clinical (on human subject), among which six are ex vivo studies [22,32,38,39,40,41] and one is an in vivo “wearable” study [32]. Therefore, implantable ISSs (i.e., ISEs) for orthopaedics are still far from clinical validation.

While Table 2 focuses on the validation environment, this information should be integrated with the target temporal benchmark for mapping the ISS technology readiness levels (TRLs). Short-term operation corresponds to hours–days, medium-term to weeks, long-term to months–years. Therefore, the device readiness level depends on its category, i.e., operational environment and duration; that is, in hours for ex vivo, in days–weeks for acute in vivo, and in months–years for chronic implantable. In light of this, the ISSs screened here for potential orthopaedic translation demonstrated a TRL 4 (i.e., technology validated in lab) for ex vivo or acute in vivo use, but a TRL 3 (i.e., experimental proof of concept) for wearable or implantable use, because of the short times of performance.

## 4. Discussion

The studies included in this review demonstrate the remarkable versatility of ion-selective sensors (ISSs) towards orthopaedics. On the one hand, the technological research on ion-selective electrodes (ISEs) has produced sensors with improved metrological performance and biocompatibility, generating their potential for implantable use. On the other hand, non-electrochemical ISSs, such as fluorescent and colorimetric probes, offer complementary advantages, including miniaturization, low-cost readings, and multiplexing, which could be particularly useful for rapid screening or point-of-care testing. Overall, these advances highlight the strong potential of ISSs to provide continuous, real-time information on ionic dynamics in musculoskeletal tissues, implants, and peri-implant environments. Indeed, there are many ions and areas of orthopaedic interest that have not been cited, but could benefit from ISSs as well, that is, every time an ion (i.e., any atom or molecule charged electronically) is crucial in the biological process. For example, strontium and boron ions were not targeted by the included papers, but they are both important for bone metabolism and against skeletal loss [78]; metal ions, like calcium, magnesium, and zinc, can directly affect the tumour microenvironment, as in bone cancer [79]; sodium and potassium ISSs can measure neural activity by extracellular measurement and thus be part of a bionic neural link for implantable prosthetics [80].

Despite these promising results, most available studies remain limited to ex vivo validation tests. Key limitations include poor reproducibility of sensor performance in complex biological fluids, instability due to biofouling or leaching of active components, and insufficient data on long-term in vivo durability. Furthermore, regulatory and biocompatibility requirements for clinical translation are rarely addressed explicitly, making it difficult to assess the feasibility of moving from the laboratory to the clinical setting. Another recurring challenge is the lack of harmonized reporting of the metrological parameters across different platforms, which hinders a direct comparison between devices.

Several technologies, originally developed for environmental monitoring or neuroscience, could be adapted to orthopaedics. For example, antifouling polymer coatings [23,45], nanostructured electrodes [25,27,58], and smart sensitive films with dual detection capabilities (pH/ROS) [45] could be integrated into orthopaedic implants to detect early infections or monitor osseointegration. Similarly, optical ISSs (fluorescence, colorimetric) tested in environmental or biochemical contexts [34,50,51,52,53,57] could inspire minimally invasive diagnostic strategies. Such cross-fertilization highlights the importance of multidisciplinary research to accelerate innovation.

A key design decision is whether to use implanted sensors, integrated directly into the prostheses or fixation devices, or external/wearable devices applied to the skin. Implanted sensors offer direct access to the peri-implant microenvironment, enabling the precise monitoring of local ion release, osseointegration, and early infections. However, they require highly biocompatible and stable materials, reliable data transmission, and face significant regulatory and surgical challenges. By contrast, external or wearable devices (e.g., for the real-time monitoring of sweat biomarkers for health or performance evaluations during physical exercise [81]) are minimally invasive, easier to replace, and suitable for systemic monitoring during rehabilitation, but suffer from reduced sensitivity to local changes and greater exposure to biological noise. From a clinical perspective, wearable sensors may be preferable for short-term or systemic monitoring, while implantable systems are more suitable for long-term, localized assessment of implant performance and complications. Another research area of interest could be to provide orthopaedic instrumentation with sensors (e.g., to reveal infections on the surgical site), with peculiar characteristics and requirements, some of them in the middle way between implantable and wearable.

### 4.1. Engineering Challenges for the Use of ISSs in Orthopaedics

Although ion-selective sensors (ISSs) show clear theoretical potential for orthopaedic applications, their translation into long-term implantable devices is currently limited by a series of system engineering constraints. These constraints concern not only the sensor chemistry but the entire architecture required for an ISS to function reliably for clinically significant periods. An implanted ISS is useless if it cannot be powered or if data cannot be extracted. None of the 44 included studies propose a power supply strategy that is truly compatible with a long-term orthopaedic implant. The reasons are structural: (i) batteries are not acceptable in implants intended to last years or decades (size, toxicity, limited lifespan); (ii) energy harvesting (piezoelectric, triboelectric, inductive) is promising, but has not yet been integrated with ion-selective platforms; (iii) passive sensors based on RFID could eliminate the need for a battery, but no study has demonstrated stable potentiometric readings through bone or deep tissues. This is not a marginal shortcoming: it is the main engineering barrier to the transition of ISSs towards real clinical applications.

In this context, joint motion-derived energy harvesting represents one of the most promising strategies for future implantable ISSs. Orthopaedic implants are inherently subjected to cyclic mechanical loading (compression, bending, torsion), which can be converted into electrical energy through piezoelectric or triboelectric elements integrated within the implant structure. Recent advances in piezoelectric energy harvesting for biomedical applications have demonstrated stable power generation under physiological deformation and compatibility with implantable formats [82]. In parallel, engineered piezoelectric biomaterials have shown efficient mechano-electrical transduction in bone-mimicking environments, supporting their integration into load-bearing orthopaedic constructs [83]. Triboelectric nanogenerators have similarly shown high stretchability, transparency, and the ability to harvest biomechanical energy from human motion [84,85]. Importantly, fully implantable piezoelectric systems have been evaluated in vivo in large-animal models, harvesting energy directly from the motion of the heart, lungs, and diaphragm at levels compatible with low-power medical electronics [86]. Although none of these systems have yet to be integrated with ion-selective sensing, they represent the most realistic pathway toward battery-free, long-term implantable ISSs.

Even with a power source, an ISS must transmit data through bone, soft tissues, and metallic materials. The studies analysed do not address wireless signal attenuation through metallic implants, the need for ultra-low power communication protocols, the trade-off between sampling frequency and energy consumption, or the risk of electromagnetic interference in the operating room or during imaging. This confirms that current ISSs are conceived as isolated sensory elements, not as complete implantable devices. An implantable ISS must withstand cyclic mechanical loads (compression, bending, torsion), fluid infiltration and corrosion, fibrotic encapsulation, and micro-movements at the bone–implant interface. None of the included studies provide hermetic sealing strategies compatible with ionic diffusion, packaging validated under orthopaedic mechanical loads, long-term corrosion resistance data, or integration pathways with real implant geometries. A sensor cannot simply be “placed” on an implant, it must be co-designed with it.

Focusing on colorimetric and fluorescence sensors, they face significant challenges in in vivo orthopaedic applications due to tissue scattering and optical attenuation. Therefore, their clinical utility currently appears to be more promising for an ex vivo analysis of peri-implant fluids or as integrated components of wearable diagnostic devices, rather than as deep implants. Colorimetric platforms operate through several mechanisms, each with specific vulnerabilities in the orthopaedic environment. Plasmonic sensors [87], which exploit the localized surface plasmon resonance (LSPR) phenomenon of noble metal nanoparticles (AuNPs, AgNPs), offer high sensitivity for detecting wear–debris ions. However, their reliability in vivo is often compromised by the formation of a “protein corona” [88], where non-specific protein adsorption causes premature nanoparticle aggregation. Conversely, complexation with organic dyes remains the most straightforward approach, but presents issues with reagent leaching and potential cytotoxicity. Emerging platforms based on nanozymes overcome these stability issues by mimicking enzymatic activities using inorganic materials; nevertheless, their catalytic performance can be altered by the fluctuating redox environment typical of peri-implant inflammatory responses. These vulnerabilities necessitate robust encapsulation strategies or the restriction of these technologies to ex vivo diagnostic tests.

Why are these challenges critical in orthopaedics? Because orthopaedic implants must function for years or decades, not for days. The most advanced case in the literature—a biodegradable Ca^2+^ sensor with a 4-day in vivo lifespan, the only example of an implantable ISS with in vivo data, but lasting only 4 days—is technically interesting, but far from clinical requirements [25]. This gap is even more pronounced in the case of non-electrochemical platforms. Many colorimetric sensors described in the literature function as irreversible dosimeters rather than continuous monitors. In addition to functional and mechanical stability, a key engineering challenge for colorimetric ISSs is the biocompatibility of their chemical components. While ionophore-based membranes for potentiometric sensors can be optimized for low cytotoxicity, colorimetric platforms often rely on indicator dyes and metallic nano-materials (e.g., silver nanoparticles or transition metal oxides) that present well-documented toxicity and leaching issues. The release of these reagents into the peri-implant environment not only leads to signal degradation over time but can also trigger localized inflammatory responses or systemic toxicity. Current strategies to mitigate this problem, such as covalent immobilization or dense encapsulation, often involve a trade-off in terms of sensor sensitivity and response time. Until zero-leaching architectures are validated, these biocompatibility risks represent a fundamental obstacle to long-term implantation, reinforcing the current preference for using these platforms in ex vivo diagnostic settings or in short-term post-operative drainage monitoring, where direct and prolonged contact with tissues is avoided. Regarding sensor reversibility, however, the reliance on external reagents, such as EDTA—as observed in some experimental setups—presents an insurmountable engineering barrier for autonomous systems. For these technologies to be viable, research must focus on reversible molecular switches and encapsulation strategies that prevent reagent leaching, which currently poses both functional and toxicological risks. Furthermore, the specific chemical vulnerabilities discussed previously (e.g., protein corona formation, dye leaching, and redox interference) generate significant ‘matrix effects’. This implies that the limits of detection (LODs) achieved in ideal, transparent laboratory buffers are rarely reproducible in vivo, where complex protein-rich synovial fluids, optical attenuation, and tissue opacity severely hinder signal acquisition and reliability. The following are missing from the literature: multi-week or multi-month implantation studies, chronic drift characterizations, data on calibration stability after fibrotic encapsulation, and analyses of the impact of protein adsorption and macrophage adhesion. This indicates that long-term stability is not simply “unresolved”, it is still unexplored under realistic orthopaedic conditions. Thus, in light of the current evidence, the only achievable outcome is short-term operation (hours–days), with improved membranes and antifouling coatings; medium-term operation (1–4 weeks) is plausible, and long-term operation requires radical innovations. These innovations include membrane chemistry (non-leachable ionophores, redox-buffered solid contacts), antifouling strategies (zwitterionic coatings, hydrogel barriers), reference-free architecture, passive telemetry or energy harvesting, and mechanically robust packaging.

Focusing on long-term stability remains a crucial and main challenge; there are still no ion-selective implantable devices validated for long-term clinical use. New research is focusing precisely on overcoming the engineering limitations, in particular, the following: (i) the use of solid-contact ISSs based on carbon materials (e.g., graphene), which eliminate the interfacial water layer and drastically reduce potential drift [89]; (ii) extreme miniaturization for sensor integration, e.g., inside the spaces of a titanium porous implant or mechanically protecting it from orthopaedic load; (iii) the development of non-leaching membranes (without component release). Although the specific in vivo data for orthopaedics are limited, the literature on low-cost electrochemical sensors in other critical sectors suggest that stability can be managed through a combination of new transducer materials and robust calibration models that take into account environmental variations (temperature, ionic interferences) [90]. Despite advances in miniaturization, long-term stability remains conditioned by the kinetics of transmembrane ionic fluxes. Recent studies have suggested that the use of solid-contact materials with high interfacial capacitance, such as MXenes or carbon nanocomposites, can rapidly stabilize the redox equilibrium, reduce the conditioning time, and minimize signal drift [91]. However, the primary engineering challenge has now shifted to the mechanical stability of the packaging [92]. As highlighted by recent studies [5], the migration of water and ions through the selective membrane affects both the conditioning time and signal stability. Reducing these fluxes allows for redox equilibrium to be achieved more quickly and improves reproducibility. In orthopaedics, the system must ensure biocompatible hermeticity under cyclic loads; the adoption of multilayer coatings (e.g., alumina/parylene) and NFC (near-field communication) telemetry systems represents the most promising solution for ‘on-demand’ monitoring that bypasses the limitations of integrated batteries and environmental degradation [93].

A recent advance particularly relevant to this discussion is the multimodal biosensing platform developed by Zulkarnine et al. [94], based on nano-corrugated graphene. This architecture achieves high electrochemical stability, eliminates the interfacial water layer, and enables multimodal sensing (electrochemical and non-electrochemical) through surface functionalization alone. These properties directly align with the design principles required for implant-integrated ISSs, reinforcing the role of graphene-based solid contacts as a promising route to long-term drift suppression. Moreover, the demonstration of combining multiple sensing modalities on a single chip is also relevant to the ISFET-based platforms discussed in Section 3.9, where hybrid architectures may offer improved robustness under complex biological conditions.

Given these optical and chemical limitations, colorimetric ISSs find their most immediate clinical application not as deep-tissue implants, but as tools for acute post-operative monitoring. By integrating these sensors into surgical drainage systems, clinicians could perform a real-time ex vivo analysis of peri-implant fluids during the critical first 48 h after surgery. This approach circumvents the engineering challenges associated with tissue opacity and long-term sensor degradation, providing a highly valuable diagnostic window for the early detection of infections. For a feasible use in routine clinical practice, colorimetric point-of-care devices should be validated on protein-rich orthopaedic biofluids that can be retrieved in a sufficient volume and in a way minimally invasive for the patient. This is the case with peripheral blood, but also with joint synovial fluid; indeed, this fluid is usually sampled in a 0.5–3 mL range, compatible with the solution volumes tested by colorimetric ISSs [11,53]. Moreover, even if synovial fluid aspiration is not very painful, it cannot be performed continuously over a period because of limited fluid regeneration and possible joint inflammation for multiple extractions [95]; therefore, it allows for clinical monitoring at intervals of months. Finally, colorimetric ISSs could reach continuous post-operative monitoring by following the recent advancements of other emerging colorimetric sensing wearable frameworks. This is the case with smart colorimetric hydrogels utilizing mechanochromic (force-sensing) properties for applications, such as evaluating joint biomechanics or surgical wound tension, and thermochromic (temperature-sensing) properties for continuous post-operative monitoring [96,97].

Biocompatibility is a necessary requirement, but not sufficient. As cited above, anti-biofouling to assure signal fidelity should be addressed as well, and the way to proceed strongly depends on the design decision, i.e., if targeting “wearable” or “implantable”. The second case is far more challenging because of the aggressive environment along with the time of exposure; the papers reviewed here have not evaluated the eventual fouling of sensors when implanted. Therefore, another study should be referenced, that of Xu and Lee (2020), which gives strategies to mitigate biofouling and the foreign body response for long-term use of implantable sensors [98]. There are state-of-the-art anti-biofouling passive (e.g., use of hydrophilic surfaces or zwitterionic polymers) and active (e.g., use of pH-responsive materials or surfactant-desorbing surfaces) approaches that should be integrated in sensor development to avoid biofouling-related failure and to pave the way for a new generation of implantable ISSs enabling innovative orthopaedic diagnostics and therapeutics.

Definitely, ISSs are still at a technology readiness level far distant from clinical standards for chronic deep implantation. This is true for sensors in general. Indeed, *Proton Intelligence*’s study is the first targeting only 6 h of use in a human clinical trial for continuous potassium monitoring in patients with kidney disease. *Eversense* produces a sensor that lasts up to 6 months for continuous glucose monitoring in diabetic patients. However, both are wearable systems. In the case of a chronic orthopaedic implant, actually the limitations are strong. Overall, the cumulative drift in the output signal can arise from reference electrode instability, liquid junction potentials, membrane degradation, and ion flux, producing a high loss in sensitivity. In the case of implanted ISSs, all the studies included in this review are strictly pre-clinical. Moreover, as previously described, the ISS with the longest time after implantation lost sensitivity already at day 5, with a drift of about 20% with respect to day 4 [25]. Therefore, implantable ISSs are distant from clinical application. At least, the recent study of Li et al. (2026), which developed a sensor for real-time in vivo monitoring of biochemical markers in synovial fluid and applied it on rats, can give some important indications [95]. In fact, the authors demonstrated a decrease in sensitivity lower than 5% after 4 weeks of implantation, validating the sensor potential for the long-term inside-joint monitoring of biochemical molecules [95].

### 4.2. Limitations of the Review Process

The searching of papers lacks specificity. The right flow would have been to start with a core question, in this case, for example, “Which ISSs are on the critical path for solving a specific orthopaedic problem?”, which instead has been output of the synthesis. However, it is the first time that the literature on ion-selective sensors has been put in relation with the orthopaedic field, thus it was preferred to start with a broad search and in-depth screening for revealing and discussing pre-clinical and clinical challenges.

## 5. Conclusions

Ion-selective sensors (ISSs) appear to be promising for diagnosing peri-implant infection, monitoring implant wear and corrosion, and for assessing bone health and metabolism. The actual level of ISSs’ development permits a clinical application, limited to ex vivo tests. While for in vivo implantable devices, the gap in pre-clinical validation is consistent.

ISSs, including ion-selective electrodes, should be designed for and tested in big animal models resembling the working conditions expected in human patients. Specifically, ISSs should satisfy the requirements of biocompatibility, anti-biofouling, low power consumption, mechanical adaptation and resistance, continuous calibration, and stable signal transmission for a long period (i.e., from hours to weeks/months). Looking at the actual technological level, possible solutions to those requirements were described, but appear at an early prototypical stage.

Therefore, before entering large animal trials, the measure of ions prioritizing anti-biofouling and self-powering devices (e.g., by passive radio frequency identification-based sensors that require no implanted battery) should move from simple PBS buffers to complex ex vivo setups resembling realistic tissues and conditions, using ex vivo synovial fluid from revision surgeries and under mechanical stress.

In conclusion, this review demonstrates that, while ISSs hold immense theoretical promise for next-generation orthopaedic care, the field is currently at technology readiness levels (TRLs) 2–3. The critical barriers to translation are not just material science, but system-level integration of power, telemetry, and long-term biostability. Without a concerted, interdisciplinary effort to address these ‘valley of death’ challenges, ISSs will remain a laboratory curiosity rather than the transformative clinical tool they have the potential to be.

## Figures and Tables

**Table 1:** Search terms used in the Scopus and Pubmed databases.

Electronic Database	Query
Scopus	TITLE-ABS-KEY (electrodes OR sensors) AND TITLE-ABS-KEY ((ion AND selective) OR ise) AND (orthopaedic OR orthopedic OR musculoskeletal OR implant OR “joint replacement”) AND PUBYEAR > 2015 AND PUBYEAR < 2026 AND (LIMIT-TO (DOCTYPE, “ar”)) AND (LIMIT-TO (LANGUAGE, “English”))
Pubmed	(((electrodes [Title/Abstract] OR sensor [Title/Abstract]) AND ((ion [Title/Abstract] AND selective [Title/Abstract]) OR ise [Title/Abstract])) AND (orthopaedic OR orthopedic OR musculoskeletal OR implant OR “joint replacement”)) Filters: English, from 2016–2025

**Table 2:** ISS studies classified by target ion–potential orthopaedic application, level of validation, sensor type (non-ISE are underlined), and pre-clinical/clinical phase (clinical in bold).

Target Ion	Target Application (Orthopaedic Relevance)	Level of Validation [Targeting ISS]			
		Non-Biological Samples	In Vitro/Ex Vivo	In Vivo Wearable	In Vivo Implantable
Ca^2+^	Bone/Muscle/Nerve metabolism; Implants (osteointegration, scaffolds); On-body measurements		pre-clinical phase [20,21,22,23]; clinical phase **[24]**		[25,26]
K^+^	Bone/Muscle/Nerve metabolism; On-body measurements	[27,28,29,30]	pre-clinical phase [23,29,31,32], clinical phase **[33]**	[32]	[26]
Na^+^	Bone/Muscle/Nerve metabolism; On-body measurements	[27,30]	pre-clinical phase [31,32]; clinical phase **[33]**	[32]	[26]
F^−^	Bone/Muscle/Nerve metabolism; Implants (osteointegration, scaffolds)	[34,35,36]	pre-clinical phase [37], clinical phase **[38,39,40,41]**		
H^+^ (pH)	Bone metabolism; Implants (osteointegration, scaffolds); Inflammation/Infection; On-body measurements		[33,42,43,44,45]		[46]
Zr^4+^	Implant (metal release)	[47]	[48]		
Ni^2+^	Implant (metal release)	[49]	[50]		
Cr^6+^	Implant (metal release)	[51,52]			
Al^3+^	Implant (metal release)	[53]			
Co^2+^	Implant (metal release)	[53]			
Mg^2+^	Implant (metal release)	[54]			
Ag^+^	Implant (metal release)	[55]			
Cu^2+^	Implant (metal release)	[56,57,58,59]	[60]		
Au^3+^	Inflammation	[61]			
Immunoglobulins	Inflammation/Infection		[62]		
PO_4_^3−^	Implant (osteointegration, scaffolds)	[63]			
NH^4+^	Bone metabolism; On-body measurements		[24,33,64]		

## Data Availability

No new data were created or analysed in this study. Data sharing is not applicable to this article.

## Supplementary Materials

The following supporting information can be downloaded at: https://www.mdpi.com/article/10.3390/bios16060302/s1, Table S1: Non-electrochemical ISSs: available performance indicators; Table S2: Main metrological parameters of the ISE developed for orthopaedic applications.
