# Supplementary material for: Ion-Selective Sensors for Orthopaedic Applications: A Systematic Review
Source: Biosensors (Basel). 2026 May 22;16(6):302. doi: 10.3390/bios16060302 (PMC13297247; doi:10.3390/bios16060302)
Supplement: Supplementary file 1 [file biosensors-16-00302-s001.zip › biosensors-4289056-supplementary.pdf]

TABLE S1

NON-ELECTROCHEMICAL ISSS: AVAILABLE PERFORMANCE INDICATORS

Ref	Ion target	Detection principle	LOD	Linear range	Response time	Selectivity / Interference	pH / Operating conditions	Notes / Orthopaedic relevance
Yang et al., 2022 [1]	Ca^{2+}, K^{+}, Na^{+}	CNT-array ion-sensitive electrode (tipo ISFET)	NA	Physiological range (Ca 1–1.5 mM; K 2.7–5 mM; Na 136–150 mM)	NA	Qualitative interference tested	NA	Sensitivity: Ca 28.86, K 48.38, Na 52.28 mV/dec
Kaewnok et al., 2025 [2]	Au^{3+}	Fluorescence (helicene probe, paper strip)	8.0 ppb	0–25 µM	~15 min	High selectivity vs 20+ ions	pH 3–11	Heavy metal detection; relevant for implant metal toxicity
Liu et al., 2024 [3]	Zr^{4+}	Colorimetric (His-AuNP dual-mode)	2.62 µM	15–70 µM	~60 min	Good selectivity vs other metals	pH 3–12	Zr^{4+} release from Zr-based implants
Yaghoubi et al., 2024 [4]	Ag^{+}	Voltammetric nanocomposite (ZIF-67/rGO)	4×10^{-11} M	10^{-10}–10^{-8} M	NA	No interference from 15+ ions	pH 6	Antimicrobial coatings; Ag^{+} toxicity monitoring
Mousavi et al., 2024[5]	Cu^{2+}	Colorimetric quinolinol probe	81.7 nM	0–50 µM	<0,01 sec	Excellent selectivity	pH 5–11	Cu^{2+} release from implants; reversible with EDTA
Singh et al., 2024 [6]	Zr^{4+}	Azomethine fluorescent probe	31.5 nM	NA	NA	Excellent selectivity	NA	Zr^{4+} detection in implant degradation
Jain et al., 2023 [7]	Cu^{2+}	Luminescent AIEgen probe	4.4–12.1 nM	NA	NA	High selectivity	pH 7.4	Cu^{2+} toxicity; implant degradation
Swami et al., 2022 [8]	F^{-}	Colorimetric AgNP-PEG sensor	0.098 mg/L	$R^2 = 0.95$	NA	NA	Stable 1 month	Fluoride in bone quality / fluorosis

Saikrithika et al., 2022 [9]	Ni^{2+}	SECM imaging (electrochemical, non-ISE)	2.08×10^{-12} g/20 µL	NA	NA	Tested vs AA, DA, UA, NADH, glucose	pH 7	Ni^{2+} release from Ni-Ti implants
Zhang et al., 2021 [10]	Ni^{2+}	Fluorescence (phenanthroline probe)	NA	NA	Fast	Exceptional selectivity	NA	Ni^{2+} release from implants
Ebrahim et al., 2020 [11]	Cr^{6+}	Fluorescent polyaniline/AgNP/GO	0.0065 mg/L	0.01–7.5 mg/L	NA	High recovery (95–99%)	NA	Cr^{6+} toxicity; implant corrosion
Bartwal et al., 2020 [12]	Al^{3+}, Co^{2+}	Fluorescent/colorimetric quinoline probes	10^{-8} M (Al^{3+}), 9.1×10^{-8} M (Co^{2+})	NA	NA	High selectivity; reversible with EDTA	pH 7.2	Metals released from implants
Li et al., 2020 [13]	pH (bone)	Fluorescent FITC-alendronate	NA	Linear 6.8–7.4	NA	NA	pH 6–12	Bone pH monitoring in vivo
Ming et al., 2019 [14]	Cr^{6+}	N-doped carbon dots (fluorescent)	1.26 nM	0.1 µM–430 µM	NA	Good repeatability	pH 3	Cr^{6+} toxicity; implant corrosion
Bao et al., 2019 [15]	NH_4^+, K^+, Ca^{2+}	ISFET flexible transistor	NH_4^+: 98 mV/dec; K^+: 104 mV/dec; Ca^{2+}: 42 mV/dec	10^{-6}–1 M (K^+, NH_4^+), 10^{-4}–1 M (Ca^{2+})	NA	Improved stability with AgCl reference	NA	Wearable/implantable FET ion sensors
Habib et al., 2019 [16]	Cu^{2+}	Optical/electrochemical chemosensor	3.08–3.64×10^{-7} M	NA	NA	High selectivity	pH 2–12	Cu^{2+} detection in implant degradation
Li et al., 2016 [17]	Cu^{2+}	Nanoporous silicon test-paper	µmol/L range	5×10^{-7}–5×10^{-6} M	NA	High selectivity	NA	Rapid Cu^{2+} detection (environmental → translational)

[1] H. Yang *et al.*, "Carbon Nanotube Array-Based Flexible Multifunctional Electrodes to Record Electrophysiology and Ions on the Cerebral Cortex in Real Time," *Adv. Funct. Mater.*, 2022, doi: 10.1002/adfm.202204794.

[2] N. Kaewnok *et al.*, "Propargylic-linked [5]helicene derivative for selective Au3+ detection in near-perfect aqueous media with applications in diverse real samples, paper test strips, and human cells," *Spectrochim. Acta - Part A Mol. Biomol. Spectrosc.*, vol. 329, no. December 2024, p. 125594, 2025, doi: 10.1016/j.saa.2024.125594.

[3] Z. Liu, Y. Yan, J. Li, W. Zhou, H. Gao, and R. Lu, “Rapid visual dual-mode detection of Zr(IV) based on l-histidine functionalized gold nanoparticles,” *Anal. Sci.*, vol. 40, no. 7, pp. 1269 – 1278, 2024, doi: 10.1007/s44211-024-00557-z.

[4] M. Yaghoubi, A. R. Zanganeh, N. Mokhtarian, and M. H. Vakili, “Nanocomposite of ZIF-67/reduced graphene oxide as sensing element for voltammetric detection of silver(I) ion: optimizing operational conditions by half-fraction central composite design,” *Monatshefte fur Chemie*, vol. 155, no. 6, pp. 561 – 571, 2024, doi: 10.1007/s00706-024-03207-2.

[5] S. Hossein Mousavi, M. Ali Zanjanchi, A. Mohammadi, B. Khalili, and H. Fallah Moafi, “A quinolinol-based colorimetric chemosensor for highly sensitive and selective detection of Cu^{2+}: Experimental and DFT studies and its application in real samples,” *J. Photochem. Photobiol. A Chem.*, vol. 451, 2024, doi: 10.1016/j.jphotochem.2024.115521.

[6] G. Singh *et al.*, “Azomethine functionalized platform for the selective detection of Zr(IV) ion, biological evaluation and potent TLR-4 inhibitor,” *J. Mol. Struct.*, vol. 1297, 2024, doi: 10.1016/j.molstruc.2023.136916.

[7] A. Jain, S. De, and P. Barman, “Salicylaldehyde-diphenyl-azine skeleton-based ESIPT-coupled AIEgens with tunable emission and applicable as highly selective and sensitive Cu2+ ion sensor,” *Dye. Pigment.*, vol. 220, 2023, doi: 10.1016/j.dyepig.2023.111769.

[8] S. Swami, A. Agarwala, V. Shrivastava, and R. Shrivastava, “Poly (ethylene glycol)-400 crowned silver nanoparticles: a rapid, efficient, selective, colorimetric nano-sensor for fluoride sensing in an aqueous medium,” *J. Chem. Sci.*, 2022, doi: 10.1007/s12039-021-02002-4.

[9] S. Saikrithika, A. Shaju, B. Dinesh, and A. S. Kumar, “In-situ scanning electrochemical microscopy interrogation on open-circuit release of toxic Ni2+ ion from Ni-containing carbon nanomaterials and nickel-hexacyanoferrate formation in physiological pH and its thiol-electrocatalysis relevance,” *Electrochim. Acta*, 2022, doi: 10.1016/j.electacta.2021.139806.

[10] G. Zhang, L. Huang, Y. Xu, S. Weng, and F. Ke, “A 1,10-phenanthroline fluorescence probe for real-time visualization of Ni2+,” *J. Iran. Chem. Soc.*, vol. 18, no. 10, pp. 2567 – 2573, 2021, doi: 10.1007/s13738-021-02215-x.

[11] S. Ebrahim, A. Shokry, M. M. A. Khalil, H. Ibrahim, and M. Soliman, “Polyaniline/Ag nanoparticles/graphene oxide nanocomposite fluorescent sensor for recognition of chromium (VI) ions,” *Sci. Rep.*, 2020, doi: 10.1038/s41598-020-70678-8.

[12] G. Bartwal, K. Aggarwal, and J. M. Khurana, “Quinoline-ampyrone functionalized azo dyes as colorimetric and fluorescent enhancement probes for selective aluminium and cobalt ion detection in semi-aqueous media,” *J. Photochem. Photobiol. A Chem.*, 2020, doi: 10.1016/j.jphotochem.2020.112492.

[13] Y. Li *et al.*, “FITC-Labeled Alendronate as an in Vivo Bone pH Sensor,” *Biomed Res. Int.*, 2020, doi: 10.1155/2020/4012194.

[14] F. Ming *et al.*, “One-step synthesized fluorescent nitrogen doped carbon dots from thymidine for Cr (VI) detection in water,” *Spectrochim. Acta - Part A Mol. Biomol. Spectrosc.*, 2019, doi: 10.1016/j.saa.2019.117165.

[15] C. Bao, M. Kaur, and W. S. Kim, “Toward a highly selective artificial saliva sensor using printed hybrid field effect transistors,” *Sensors Actuators, B Chem.*, 2019, doi: 10.1016/j.snb.2019.01.062.

[16] A. Habib, A. Saeed, G. Shabir, and S. Habib, “Optically selective and electrochemically active chemosensors for Cu (II) ions based on a skeleton of 2-(benzylideneamino)-4,5,6,7-tetrachloro-3’,6’-dihydroxyspiro-[isoindoline-1, 9’-xanthen]-3-one,” *Spectrochim. Acta - Part A Mol. Biomol. Spectrosc.*, vol. 208, pp. 185–197, 2019, doi: 10.1016/j.saa.2018.10.002.

[17] S. Li *et al.*, “An Innovative Metal Ions Sensitive ‘Test Paper’ Based on Virgin Nanoporous Silicon Wafer: Highly Selective to Copper(II),” *Sci. Rep.*, 2016, doi: 10.1038/srep36654.

TABLE S2

MAIN METROLOGICAL PARAMETERS OF THE ISE DEVELOPED FOR ORTHOPAEDIC APPLICATIONS.

**Rif**	**Ione target**	**Sensor type/ configuration**	**Sensitivity**	**LOD**	**Linear range**	**Response time (t90)**	**Selectivity/Interferents**	**Stability /Drift**	**pH range**	**Reproducibility**
[1]	Ca^{2+}	Biodegradable fiber Ca^{2+} sensor (BFCS) (ionophore covalently bound)	21.65 mV lg−1 [Ca^{2+}],	from 6.92 to 10 mM	$R2 = 0.998$	1.2 s	excellent selectivity	It can monitor the fluctuations of Ca^{2+} in a 4-day lifespan in vivo and biodegrade in 4 weeks	NA	≈97%
[2]	K^+	Inkjet-printed ASS-ISE (PVC + graphene additive) - potentiometric	linear sensitivity of 58.7 mV/decades (for K)	NA	detection range from 0 to 400 mV with a resolution of 0.1 mV; linearity uncertainty of 2.9 mV	K WE with the largest area exhibits better potential stability of ~0.3 mV and faster response time of 15 s	Upon the introduction of interfering ions, there is only a potential shift of 2.5 mV for K^+;	potential variations of 0.6 mV, recording data for a 3-min duration	NA	NA
	Na^+		sensitivity of 57.1 mV/decade (for Na);			NA	potential shift, i.e., 3.8 mV after the addition of interfering ions for Na			
[3]	K^+	ISE with BME-44 ionophore covalently attached to silicone membranes	54.1 ± 7.1	4 ± 1	NA	NA	BME-44 derivative (7) on octane-thiol–coated gold electrodes shows higher K^+ over Na^+ selectivity ($\log C < -4.05$) than mobile BME-44 (-3.54 ± 0.05).	NA	NA	NA

[4]	Na^+,	3D printed IoT-controlled robot for rapid prototyping of ISEs	58.2 ± 2.6 mV/decade	1 × 10− 5 M	10− 3 M-10− 1 M Na^+, R2 = 0.9916 (only for Na^+)	11 sec for both	common interfering metabolites including Mg^{2+} and Ca^{2+}.	for 3.5 h and, it was found that there were 0.3 mV/h,	pH: 2.0–10.0, Na^+-ISE showed stable potentiometric response from pH 4.0 to 9.0, and 9 mV and 4 mV drift in the pH range of 9.0–10.0 for 10−2 M and 10−3 M NaCl, respectively	NA
	K^+		56.1 ± 0.7 mV decade^{-1}		NA			0.5 mV/h and 1.2 mV/h change in potentiometric response, respectively, showing excellent stability	NA	
[5]	Cu^{2+}	(L1: Schiff base ligands 2-(2-hydroxyphenylimino)carboxythiophene; L2: 1,2-di(2-carboxythiopheneimino] benzene	29.10 (for L1) and 29.28 (for L2) mVdecade-1 for Cu(II) ion	L1: $1×10^{−7}$ M, L2: $5.28×10^{−8}$ M	L1: 1.18*10-7 – 1.00*10-2 M; L2: 7.91*10-8 – 1.00*10-2 M	about 2 s	good selectivity toward copper(II) ion in comparison to other common cations.	good stability	pH range 2.5–5.8.	NA
[6]	K^+	IrO_2 microprobe anti-biofouling	~Nernstian-like; K^+/Na^+ ≈ 10:1	<1 K^+ ion detection (sub-nM)	NA	NA	high (10× vs Na^+)	Over a period of 30 min, the drift in OCP is 3 mV.	NA	NA

[7]	Ca^{2+} (membranesensors)	self-sterilizing polymeric membrane (6-chloroindole release)	the potentiometric slope decreases slightly with 6-Cl indole content up to 1.0 wt %, and more markedly at higher contents, especially at elevated Ca^{2+} levels	NA	10−4 −10−1 M	After biofoulant exposure, control Ca^{2+}-ISEs show a markedly longer response time, whereas the 6-Cl indole-doped sensor remains unchanged	the major cations in seawater (i.e., K^+, Na^+, and Mg^{2+}) were selected as the interfering ions	45 days	NA	On 6-Cl indole-doped Ca^{2+}-ISMs, bacterial survival ranges from 37.4% to 50.0% over the first 13 days, then gradually increases, indicating reduced response stability.
[8]	K^+, H^+, Ca^{2+}, Na^+, NH_4^+	Ionophore-based membranes; cytotoxicity study	59.38 ± 0.05 and 54.52 ± 0.22 mV for three electrodes based on valinomycin and mutacin	10−3.9 expressed in potassium activity	from 10−3 to 10−1 expressed in potassium activity	10-30 sec	Na^+ interference was evident from the selectivity coefficients (log K_k,Na = −0.75 ± 0.06 vs. −4.20 ± 0.52 for mutacin and valinomycin)	NA	NA	NA
[9]	NH_4^+ (urea biosensor)	dendritic polypyrrole Ag nanocomposite urea biosensor (ammonium ISE)	~57 mV/decade	good	NA	40 and 50 s	NA	10 days	pH: 7	NA
[10]	ROS sensor (H_2O_2, ClO^-)	Polythiophene-porphyrin con Fe^{2+}, rivestito con poly(2-methyl-2-oxazoline) (non-biofouling layer), su GC electrode	poly-3TTP-Cu: 13.5 mV/dec (50 nM–1 µM); Poly-3TTP-Fe: 31.8 mV/dec (50 nM–10 µM); with BSA: 12.8 mV/dec; with catalase: 44.4 mV/dec	~50 nM	50 nM – 10 µM (biologically relevant)	~15 min to achieve balance	no interference from BSA; no significant response to ClO^- (except concentrations >10 µM, which are non-physiological)	robust, reversible, biocompatible film thanks to the NBL coating	NA	NA

	pH sensor	Polyaniline + poly(2-methyl-2-oxazoline) su lega Ti-6Al-4V	–59.6 mV/pH (Nernstian)	NA	Concentration range: 2000 × 105 CFU/mL to 6000 × 105 CFU/mL	3 min	tested with BSA → unchanged response (linear, slope –84.4 mV/pH in the presence of proteins)	high reproducibility, suitable for use as a maintenance-free implantable sensor	pH: 5–8 (pH sensor)	good reproducibility of 8.3 mV; (standard potential E_0 = 558.9 mV)
[11]	Ca^{2+}, K^+, Na^+	CNT array flexible multifunctional electrode (ECoG + ion sensors)	28.86 Ca^{2+}, 48.38 K^+, and 52.28 Na^+ mV dec^{-1}	NA	1.0–1.5 mm Ca^{2+}, 2.7–5.0 mm K^+, and 136–150 mm Na^+	NA	Mg^{2+} (for all sensors), Ca^{2+} (only for K^+ and Na^+ sensors), K^+ (only for Ca^{2+} and Na^+ sensors), Na^+ (only for K^+ and Ca^{2+} sensors)	stabile ion-sensing	NA	reproducibility with a RSD of sensitivity lower than 2.2% among different electrodes

[1] S. Yu *et al.*, “A Biodegradable Fiber Calcium Ion Sensor by Covalently Bonding Ionophores on Bioinert Nanoparticles,” *Adv. Healthc. Mater.*, vol. 13, no. 22, 2024, doi: 10.1002/adhm.202400675.

[2] K.-L. Tsou and Y.-T. Cheng, “Miniaturized inkjet-printed flexible ion-selective sensing electrodes with the addition of graphene in PVC layer for fast response real-time monitoring applications,” *Talanta*, vol. 275, 2024, doi: 10.1016/j.talanta.2024.126107.

[3] B. D. Spindler, X. V Chen, K. I. Graf, P. Bühlmann, and A. Stein, “Potassium Ion-Selective Electrodes with BME-44 Ionophores Covalently Attached to Condensation-Cured Silicone Membranes,” *Langmuir*, 2024, doi: 10.1021/acs.langmuir.4c01726.

[4] T. Ozer, I. Agir, and C. S. Henry, “Rapid prototyping of ion-selective electrodes using a low-cost 3D printed internet-of-things (IoT) controlled robot,” *Talanta*, 2022, doi: 10.1016/j.talanta.2022.123544.

[5] S. M. Saadeh, H. M. Abu Shawish, and M. Y. Abu Foul, “Lowering detection limits of copper(II)-selective carbon paste electrodes using an SNO- and an SNNS- Schiff base ligands,” *Sensors Int.*, vol. 3, 2022, doi: 10.1016/j.sintl.2021.100151.

[6] K. Wildner *et al.*, “Iridium oxide-based potassium sensitive microprobe with anti-fouling properties,” *IEEE Sens. J.*, 2020, doi: 10.1109/JSEN.2020.3003040.

[7] F. Mirjalili, S. Manafi, and F. Lotfi, “Examination of morphology, degradation and biocompatibility of fluorapatite–forsterite nanocomposite,” *Ceram. Int.*, vol. 46, no. 13, pp. 21256–21267, 2020, doi: 10.1016/j.ceramint.2020.05.216.

[8] R. Cánovas, S. Padrell Sánchez, M. Parrilla, M. Cuartero, and G. A. Crespo, “Cytotoxicity Study of Ionophore-Based Membranes: Toward On-Body and in Vivo Ion Sensing,” *ACS Sensors*, vol. 4, no. 9, pp. 2524–2535, 2019, doi: 10.1021/acssensors.9b01322.

[9] A. K. Pandey, P. C. Pandey, N. R. Agrawal, and I. Das, “Synthesis and characterization of dendritic polypyrrole silver nanocomposite and its application as

a new urea biosensor," *J. Appl. Polym. Sci.*, 2018, doi: 10.1002/app.45705.

[10] I. Ivanko, E. Tomšík, and M. Hrubý, "Development of Smart Potentiometric Sensors Covered with Non-biofouling Layer for Detection of Early Stages of Inflammatory Processes Around Joint Replacements," *Chem. List.*, 2023, doi: 10.54779/chl20230425.

[11] H. Yang *et al.*, "Carbon Nanotube Array-Based Flexible Multifunctional Electrodes to Record Electrophysiology and Ions on the Cerebral Cortex in Real Time," *Adv. Funct. Mater.*, 2022, doi: 10.1002/adfm.202204794.
